# Dynamic Frequency-Decoupled Refinement Network for Polyp Segmentation

**DOI:** 10.3390/bioengineering12030277

**Published:** 2025-03-11

**Authors:** Yao Tong, Jingxian Chai, Ziqi Chen, Zuojian Zhou, Yun Hu, Xin Li, Xuebin Qiao, Kongfa Hu

**Affiliations:** 1 School of Artificial Intelligence and Information Technology, Nanjing University of Chinese Medicine, Nanjing 210023, China; yaotong@njucm.edu.cn (Y.T.); 20221130@njucm.edu.cn (J.C.); huy@njucm.edu.cn (Y.H.); 2 Jiangsu Province Engineering Research Center of TCM Intelligence Health Service, Nanjing University of Chinese Medicine, Nanjing 210023, China; qiaoxb@njmu.edu.cn; 3 Vanke School of Public Health, Tsinghua University, Beijing 100084, China; chenzq21@mails.tsinghua.edu.cn; 4 College of Computer Science and Software Engineering, Hohai University, Nanjing 211100, China; li-xin@hhu.edu.cn; 5 School of Elderly Care Services and Management, Nanjing University of Chinese Medicine, Nanjing 210023, China

**Keywords:** polyp segmentation, neural network, attention mechanism, colonoscopy image

## Abstract

Polyp segmentation is crucial for early colorectal cancer detection, but accurately delineating polyps is challenging due to their variations in size, shape, and texture and low contrast with surrounding tissues. Existing methods often rely solely on spatial-domain processing, which struggles to separate high-frequency features (edges, textures) from low-frequency ones (global structures), leading to suboptimal segmentation performance. We propose the Dynamic Frequency-Decoupled Refinement Network (DFDRNet), a novel segmentation framework that integrates frequency-domain and spatial-domain processing. DFDRNet introduces the Frequency Adaptive Decoupling (FAD) module, which dynamically separates high- and low-frequency components, and the Frequency Adaptive Refinement (FAR) module, which refines these components before fusing them with spatial features to enhance segmentation accuracy. Embedded within a U-shaped encoder–decoder framework, DFDRNet achieves state-of-the-art performance across three benchmark datasets, demonstrating superior robustness and efficiency. Our extensive evaluations and ablation studies confirm the effectiveness of DFDRNet in balancing segmentation accuracy with computational efficiency.

## 1. Introduction

Colorectal cancer (CRC) is one of the leading causes of cancer-related deaths worldwide [1,2,3,4,5,6]. Early detection through colonoscopy significantly reduces mortality, and automatic polyp segmentation plays a crucial role in assisting clinicians by improving diagnostic accuracy and efficiency. However, precise polyp segmentation remains a challenging task due to variations in polyp size, shape, texture, and location, as well as the inherently low contrast between polyps and surrounding mucosal tissues [7,8]. Traditional methods based on handcrafted features have demonstrated limited robustness and generalizability, failing to adapt to diverse polyp appearances [9,10,11].

Deep learning techniques, particularly convolutional neural networks (CNNs), have significantly advanced polyp segmentation by enabling hierarchical feature extraction [12]. U-Net [13], with its encoder–decoder structure and skip connections, has served as a foundational model for medical image segmentation. Extensions such as UNet++ [14], ResUNet [15], PraNet [16], and LDNet [17] have further enhanced segmentation accuracy by improving feature representation and model robustness. Despite these improvements, CNN-based models often struggle with boundary ambiguity and fail to capture fine-grained polyp details, which are critical for clinical decision making [18,19].

To address CNNs’ limitations in modeling long-range dependencies, transformer-based models have been introduced for medical image segmentation [20,21,22,23]. TransUNet [24] integrates convolutional layers with transformers to enhance global context modeling while preserving fine-grained spatial features. TransFuse [25] employs a dual-branch structure combining CNNs and transformers, using a BiFusion module to merge spatial and global features. Other transformer-based architectures such as SSFormer [26], ColonFormer [27], and Polyp-PVT [28] leverage hierarchical multi-scale attention to capture global semantic dependencies and enhance boundary representations. More recent approaches, including MGCBFormer [29] and MIA-Net [30], focus on precise boundary modeling using multiscale grid priors and hybrid CNN-transformer architectures. Despite their effectiveness, most transformer-based models operate solely in the spatial domain, overlooking the potential benefits of frequency-domain analysis. This omission limits their ability to fully exploit the complementary advantages of spatial and frequency representations, particularly when segmenting texture-rich polyps with indistinct boundaries.

Given these limitations, the following two key challenges persist in polyp segmentation:

1. Existing methods struggle to effectively separate high-frequency components (e.g., edges and textures) from low-frequency components (e.g., global shapes and structural coherence). This often results in imprecise boundaries and incomplete segmentations, particularly for polyps with irregular morphology or highly textured surfaces.
2. Most segmentation models primarily extract features in the spatial domain, neglecting the complementary insights that frequency-domain representations can provide. The frequency domain is particularly effective in capturing both local details and global patterns, making it crucial for addressing low-contrast and visually ambiguous polyp regions.

These challenges highlight a critical gap in existing segmentation frameworks, underscoring the need for a model that dynamically decouples and optimizes high- and low-frequency information while effectively leveraging both spatial and frequency-domain features.

To overcome these challenges, we propose the Dynamic Frequency-Decoupled Refinement Network (DFDRNet), a novel framework designed for polyp segmentation. DFDRNet introduces a specialized DFDR module that dynamically refines both high- and low-frequency features and fuses them with spatial representations within a U-shaped encoder–decoder architecture. The proposed approach offers the following key contributions:

1. We introduce the DFDR module, consisting of Frequency Adaptive Decoupling (FAD) and Frequency Adaptive Refinement (FAR) submodules. FAD utilizes frequency-domain analysis via Fast Fourier Transform (FFT) to adaptively separate high-frequency (edge-focused) and low-frequency (structure-focused) components, while FAR refines these features through edge enhancement and global consistency modeling. This enables precise boundary delineation and improved segmentation robustness.
2. DFDRNet incorporates DFDR modules at multiple levels of the encoder–decoder structure, allowing for progressive refinement of spatial and frequency-domain features across scales. This hierarchical design enhances the network’s ability to capture fine-grained polyp details while preserving global structural coherence.
3. We validate DFDRNet on three widely used polyp segmentation datasets, demonstrating superior performance over CNN-based and transformer-based methods. Extensive ablation studies further confirm the effectiveness of DFDR in dynamically optimizing frequency components and improving segmentation accuracy while maintaining computational efficiency.

## 2. Related Works

### 2.1. Traditional Methods for Polyp Segmentation

Early approaches to polyp segmentation predominantly relied on handcrafted feature extraction and classical image processing techniques [31,32]. These methods employed edge detection, threshold-based segmentation, and morphological operations to delineate polyp boundaries. For example, Xia et al. [33] proposed a segmentation pipeline that first identified a preliminary region of interest (pROI) using a modified Hough transform. The background was then removed, followed by a two-step segmentation process involving relaxation-based region segmentation and a refinement step to merge redundant segments based on color differences in the CIE color space. Similarly, Wang et al. [34] developed a computer-aided detection (CAD) system that analyzed both local and global geometric features of the colon wall to detect colorectal polyps. Their approach leveraged texture and morphological characteristics to enhance detection accuracy while employing an elliptical polyp model to refine candidate regions. Jerebko et al. [35] further introduced a curvature-based technique that extracted symmetry features to improve polyp differentiation from surrounding tissues.

Despite their contributions, traditional approaches faced significant limitations. The high variability in polyp size, shape, and texture posed challenges for rule-based segmentation methods, leading to suboptimal performance in complex clinical scenarios [36,37,38,39]. Additionally, these methods were often sensitive to noise, illumination changes, and variations in colonoscopy imaging conditions. To address these shortcomings, researchers explored machine learning-based techniques, including support vector machine (SVM) [40], random forest [41], and k-nearest neighbor (KNN) classifiers [42], which improved polyp detection by learning discriminative features from labeled data. However, these models still heavily relied on handcrafted features, limiting their adaptability to unseen variations in polyp morphology and background clutter.

With the advent of deep learning, particularly convolutional neural networks (CNNs), a paradigm shift occurred, enabling automated feature extraction and significantly improving segmentation accuracy [43]. This transition marked the decline of traditional feature-engineering methods in favor of data-driven approaches that could learn robust feature representations directly from images.

### 2.2. Deep Learning Methods for Polyp Segmentation

Deep learning has significantly advanced polyp segmentation, leveraging both convolutional neural networks (CNNs) and transformer-based architectures to improve accuracy, robustness, and generalization in medical image analysis [44,45,46]. Traditional CNN-based approaches, such as U-Net [13], have been widely adopted due to their encoder–decoder architecture with skip connections, enabling efficient feature extraction while preserving spatial details. Further enhancements, such as UNet++ [14] and ResUNet [15], introduced nested skip connections and residual learning to improve feature propagation and model stability. However, despite these advancements, CNN-based models often struggle with segmenting polyps that exhibit indistinct boundaries and significant morphological variations, leading to segmentation errors in challenging cases.

To address these limitations, attention mechanisms have been incorporated into CNN architectures to enhance feature representation. FAENet [47] utilizes frequency-aware attention to refine spatial and frequency-domain features, improving polyp boundary delineation. Similarly, CAFE-Net [48] introduces cross-attention mechanisms to strengthen multi-scale feature interactions, enhancing segmentation robustness. Meanwhile, CTNet [49] employs contrastive learning with transformers to model long-range dependencies, improving the contrast between polyps and surrounding tissues. These methods demonstrate that attention-based mechanisms significantly enhance polyp segmentation performance by refining feature extraction and improving boundary precision.

Building upon the success of attention mechanisms, transformer-based architectures have emerged as a powerful alternative to CNNs for medical image segmentation. Vision Transformer (ViT) [50] pioneered the use of self-attention in computer vision, treating image patches as tokens and modeling long-range dependencies through multi-head self-attention. However, ViT lacks inherent inductive biases found in CNNs, making it less efficient for dense prediction tasks such as segmentation. Swin Transformer [21] addressed this limitation by introducing a hierarchical attention mechanism with shifted windows, improving computational efficiency while preserving both local and global contextual information. Expanding on this, SwinE-Net [51] integrated Swin Transformer with convolutional layers, combining the strengths of both architectures to enhance polyp segmentation accuracy.

The success of transformers in vision tasks has led to their widespread adoption in polyp segmentation. Polyp-Mamba [52] replaces conventional convolutional and self-attention mechanisms with a Mamba-based representation, enabling efficient feature modeling across spatial scales. PRCNet [53] introduces parallel reverse convolutional attention mechanisms to capture both local and global contextual cues, improving segmentation accuracy. Meanwhile, BUA-Net [54] incorporates a boundary uncertainty-aware framework, refining segmentation maps by dynamically adjusting to regions with high uncertainty, thereby reducing false positives and negatives.

A growing trend in polyp segmentation is the integration of hybrid vision transformer models, which combine CNNs and transformers to leverage both local and global feature representations. Polyp-LVT [55] and XBound-Former [56] utilize vision transformers to enhance long-range dependencies, achieving state-of-the-art performance on multiple benchmarks. Goceri et al. [57] further advanced this paradigm by introducing a hybrid vision transformer with a specialized loss function, effectively addressing segmentation challenges in medical imaging. Despite these advancements, most transformer-based models rely solely on spatial-domain processing, overlooking the complementary benefits of frequency-domain information. Our proposed DFDRNet overcomes this limitation by dynamically integrating spatial and frequency-domain processing, ensuring robust segmentation with precise boundary refinement.

Although these advancements have significantly improved segmentation accuracy, many existing models primarily focus on spatial-domain processing without explicitly leveraging frequency-domain features. Recent works have explored frequency-based enhancements: FcaNet [58] introduces a frequency-channel attention mechanism, and FreqFormer [59] incorporates frequency-domain transformers to enhance boundary precision. However, these approaches often treat frequency and spatial features separately rather than optimizing their interactions jointly.

To address these limitations, our proposed DFDRNet introduces a novel Dynamic Frequency-Decoupled Refinement (DFDR) module, which explicitly decouples and refines high- and low-frequency components while integrating them into spatial feature processing. Unlike prior methods, DFDRNet dynamically balances local texture information (HF) and global structural coherence (LF), ensuring superior segmentation accuracy, particularly in challenging polyp detection scenarios.

## 3. Method

### 3.1. Overview

**Figure 1:**
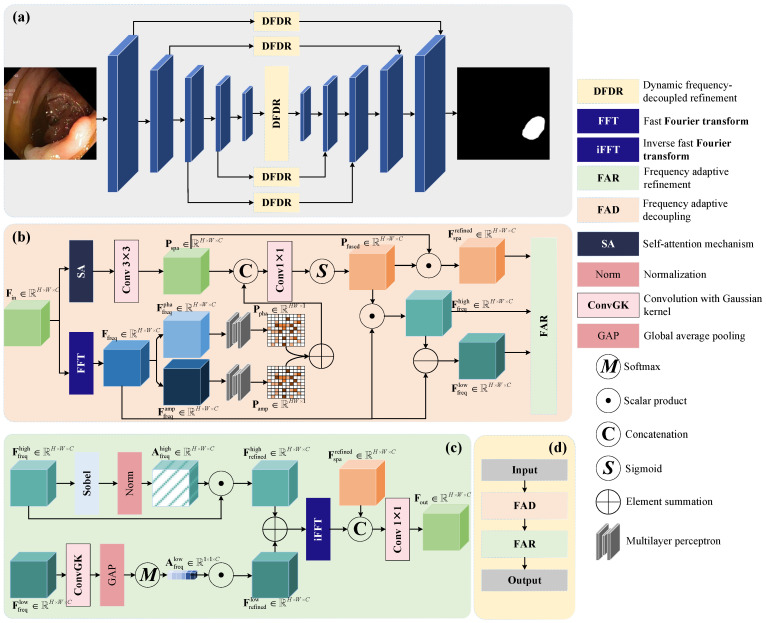
Overview of the proposed DFDRNet. (**a**) The overall architecture of DFDRNet, which follows a U-shaped encoder–decoder structure and integrates dynamic frequency decoupling at multiple stages. (**b**) The pipeline of the Frequency Adaptive Decoupling (FAD) module, where input features are decomposed into high-frequency and low-frequency components via Fast Fourier Transform (FFT) and processed independently. (**c**) The pipeline of the Frequency Adaptive Refinement (FAR) module, which enhances frequency components through amplitude-phase decomposition and selective refinement. (**d**) The DFDR module, which integrates FAD and FAR, refining both spatial and frequency-domain features before fusing them back into the segmentation network to improve boundary precision and structural consistency.

The proposed DFDRNet architecture, as illustrated in Figure 1a, follows a U-shaped encoder–decoder paradigm designed to effectively integrate spatial and frequency-domain features. This integration aims to enhance polyp segmentation accuracy by leveraging complementary information from both domains. The core innovation of DFDRNet is the DFDR module, which is strategically embedded across multiple stages of the encoder–decoder network. This module employs frequency-domain analysis to decouple input features into high-frequency (edge and texture details) and low-frequency (global structural information) components. These components undergo selective refinement before being fused with spatial features to ensure accurate boundary delineation while preserving global contextual information.

The encoder, built upon ResNet50, extracts multi-scale spatial features from the input image. These features serve as the foundation for both frequency-domain analysis and spatial-domain refinement. Specifically, at each stage of the encoder and decoder, the DFDR module decomposes features into distinct frequency components, processes them independently through adaptive filtering mechanisms, and subsequently fuses them to enhance segmentation accuracy. The hierarchical application of DFDR modules across multiple network layers progressively improves feature quality, allowing the network to preserve both local details and global structural consistency.

Furthermore, the decoder reconstructs the segmentation map by progressively refining the extracted features. This refinement involves adaptive feature weighting, guided attention, and cross-domain fusion, ensuring that critical polyp boundaries are well preserved while suppressing irrelevant noise. By dynamically refining both spatial and frequency features, DFDRNet effectively mitigates common challenges in polyp segmentation, such as boundary ambiguity, low contrast, and structural inconsistencies. The proposed architecture is extensively validated on multiple benchmark datasets, demonstrating its robustness and effectiveness in medical image segmentation.

### 3.2. Details of DFDR Module

As illustrated in Figure 1d, the DFDR module is divided into two sequential stages: the Frequency Adaptive Decoupling (FAD) module and the Frequency Adaptive Refinement (FAR) module, shown in Figure 1b and Figure 1c, respectively. The FAD module is responsible for dynamically decomposing input features into high-frequency and low-frequency components, while the FAR module refines these decomposed components to enhance segmentation performance. The detailed operations of each module are described below.

#### 3.2.1. Frequency Adaptive Decoupling (FAD)

The FAD module receives an input feature map $F_{\mathrm{in}}\in R^{H\times W\times C}$. This feature map is processed in two parallel branches: the spatial processing branch and the frequency processing branch.

Spatial Processing Branch: The input feature map undergoes a self-attention (SA) operation followed by a 3×3 convolution, producing an enhanced spatial feature representation, as follows:

(1) $$P_{\mathrm{spa}}={\mathrm{Conv}}_{3\times 3}\left(\mathrm{SA}\left(F_{\mathrm{in}}\right)\right),\;P_{\mathrm{spa}}\in R^{HW\times C}.$$

This representation captures spatial contextual information, enabling the model to preserve fine-grained image details.

Frequency Processing Branch: The input feature map is transformed into the frequency domain using a 2D Fast Fourier Transform (FFT), as follows:

(2) $$F_{\mathrm{freq}}=\mathrm{FFT}\left(F_{\mathrm{in}}\right).$$

The resulting frequency-domain representation is then separated into phase and amplitude components, as follows:

(3) $$F_{\mathrm{freq}}^{\mathrm{pha}},F_{\mathrm{freq}}^{\mathrm{amp}}\in R^{H\times W\times C}.$$

These components are independently processed using multiyear perceptrons (MLPs) to extract phase and amplitude-specific feature embeddings, as follows:

(4) $$P_{\mathrm{pha}}=\mathrm{MLP}\left(F_{\mathrm{freq}}^{\mathrm{pha}}\right),\;P_{\mathrm{amp}}=\mathrm{MLP}\left(F_{\mathrm{freq}}^{\mathrm{amp}}\right).$$

The two embeddings are combined to generate the overall frequency context representation, as follows:

(5) $$P_{\mathrm{freq}}=P_{\mathrm{pha}}+P_{\mathrm{amp}}.$$

Finally, the spatial and frequency representations are concatenated and passed through a 1×1 convolution and sigmoid activation, as follows:

(6) $$P_{\mathrm{fused}}=\sigma \left({\mathrm{Conv}}_{1\times 1}\left(\mathrm{Concat}\left(P_{\mathrm{spa}},P_{\mathrm{freq}}\right)\right)\right).$$

This fused representation is used to dynamically decouple high- and low-frequency features, a follows:

(7) $$F_{\mathrm{freq}}^{\mathrm{high}}=P_{\mathrm{fused}}⊙F_{\mathrm{freq}},\;F_{\mathrm{freq}}^{\mathrm{low}}=F_{\mathrm{freq}}-F_{\mathrm{freq}}^{\mathrm{high}}.$$

#### 3.2.2. Frequency Adaptive Refinement (FAR)

The FAR module refines the high-frequency and low-frequency features obtained from FAD.

High-Frequency Refinement: To suppress noise and enhance edge details, the high-frequency feature map undergoes a Sobel filtering operation followed by normalization, as follows:

(8) $$A_{\mathrm{freq}}^{\mathrm{high}}=\mathrm{Norm}\left(\mathrm{Sobel}\left(F_{\mathrm{freq}}^{\mathrm{high}}\right)\right).$$

This weight matrix is applied to the high-frequency features, as follows:

(9) $$F_{\mathrm{refined}}^{\mathrm{high}}=A_{\mathrm{freq}}^{\mathrm{high}}⊙F_{\mathrm{freq}}^{\mathrm{high}}.$$

Low-Frequency Refinement: The low-frequency components are smoothed using a Gaussian convolution, followed by global average pooling (GAP) and a Softmax-based attention mechanism, as follows:

(10) $$A_{\mathrm{freq}}^{\mathrm{low}}=\mathrm{Softmax}\left(\mathrm{GAP}\left(F_{\mathrm{freq}}^{\mathrm{low}}\right)\right).$$

The refined low-frequency features are computed as follows:

(11) $$F_{\mathrm{refined}}^{\mathrm{low}}=A_{\mathrm{freq}}^{\mathrm{low}}⊙F_{\mathrm{freq}}^{\mathrm{low}}.$$

Finally, the refined high- and low-frequency features are summed and transformed back into the spatial domain using an inverse FFT, as follows:

(12) $$F_{\mathrm{out}}={\mathrm{Conv}}_{1\times 1}\left(\mathrm{Concat}\left(F_{\mathrm{spa}}^{\mathrm{refined}},\mathrm{iFFT}\left(F_{\mathrm{refined}}^{\mathrm{freq}}\right)\right)\right).$$

The DFDR module thus effectively refines spatial and frequency features, enabling DFDRNet to achieve high-precision polyp segmentation.

## 4. Experiments

### 4.1. Datasets

To evaluate the effectiveness of our proposed method, we conduct experiments on the following three publicly available polyp segmentation datasets: Kvasir-SEG [60], CVC-ClinicDB [61], and CVC-ColonDB [62]. To ensure a fair and robust assessment, we adopt a standard dataset split ratio of 60% for training, 20% for validation, and 20% for testing. This 6:2:2 division is particularly well suited for relatively small datasets, as it balances sufficient training data with adequate validation and testing samples, allowing for a comprehensive evaluation of the model’s generalization ability [63]. This splitting strategy is widely adopted in medical image analysis to ensure reliable model performance assessment. Additionally, we apply data augmentation techniques, including random flipping, cropping, and intensity variations, to enhance model robustness and mitigate overfitting.

#### 4.1.1. Kvasir-SEG

**Figure 2:**
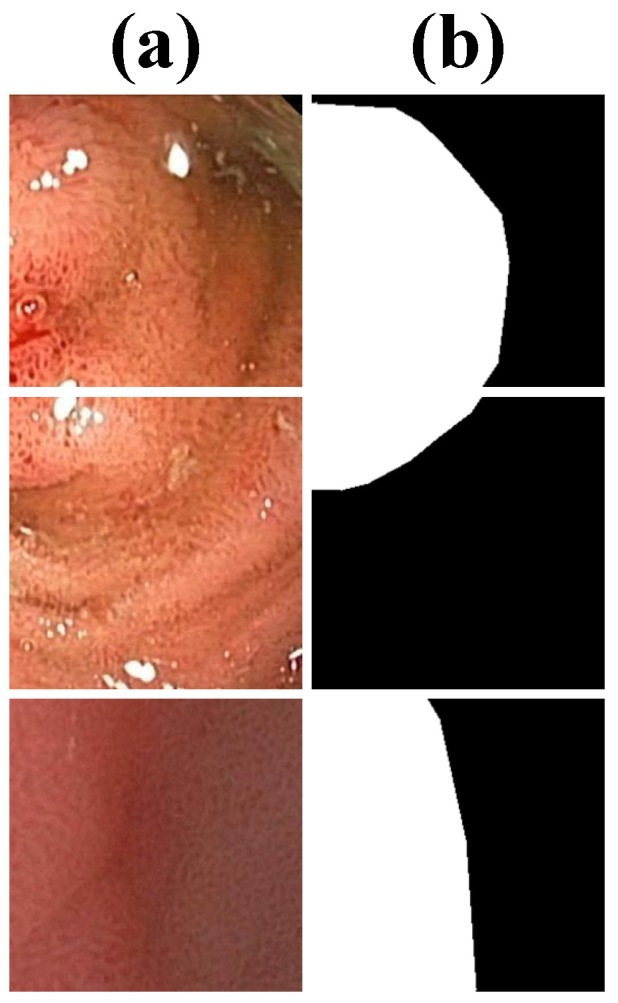
Samples from Kvasir-SEG, including (**a**) images and (**b**) labels.

Kvasir-SEG is a widely used dataset that consists of 1000 polyp images obtained from real-world colonoscopy examinations, each accompanied by pixel-wise segmentation annotations. The dataset includes a diverse range of polyp sizes, shapes, and textures, making it a comprehensive benchmark for evaluating segmentation models. As part of the Kvasir project, it plays a crucial role in advancing research on automated gastrointestinal disease detection and polyp segmentation. Several samples are visualized in Figure 2.

#### 4.1.2. CVC-ClinicDB

**Figure 3:**
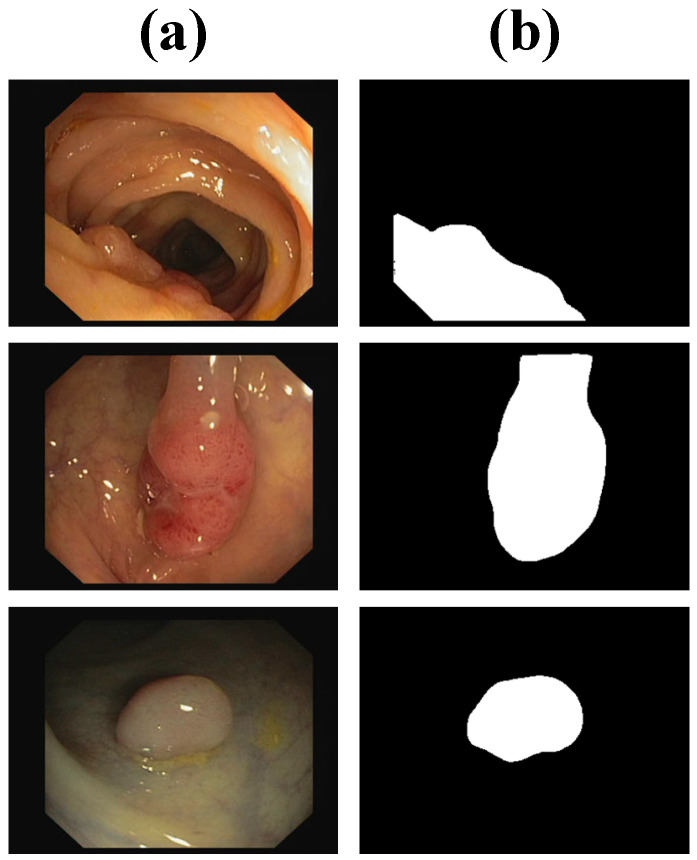
Samples from CVC-ClinicDB, including (**a**) images and (**b**) labels.

CVC-ClinicDB comprises 612 high-resolution colonoscopy images, each annotated with manually delineated polyp boundaries. The dataset captures polyps of varying morphological characteristics and is collected under diverse imaging conditions, including variations in lighting and motion. Due to its high-quality annotations and challenging cases, CVC-ClinicDB is frequently used to benchmark the accuracy and robustness of polyp segmentation algorithms. Several samples are visualized in Figure 3.

#### 4.1.3. CVC-ColonDB

**Figure 4:**
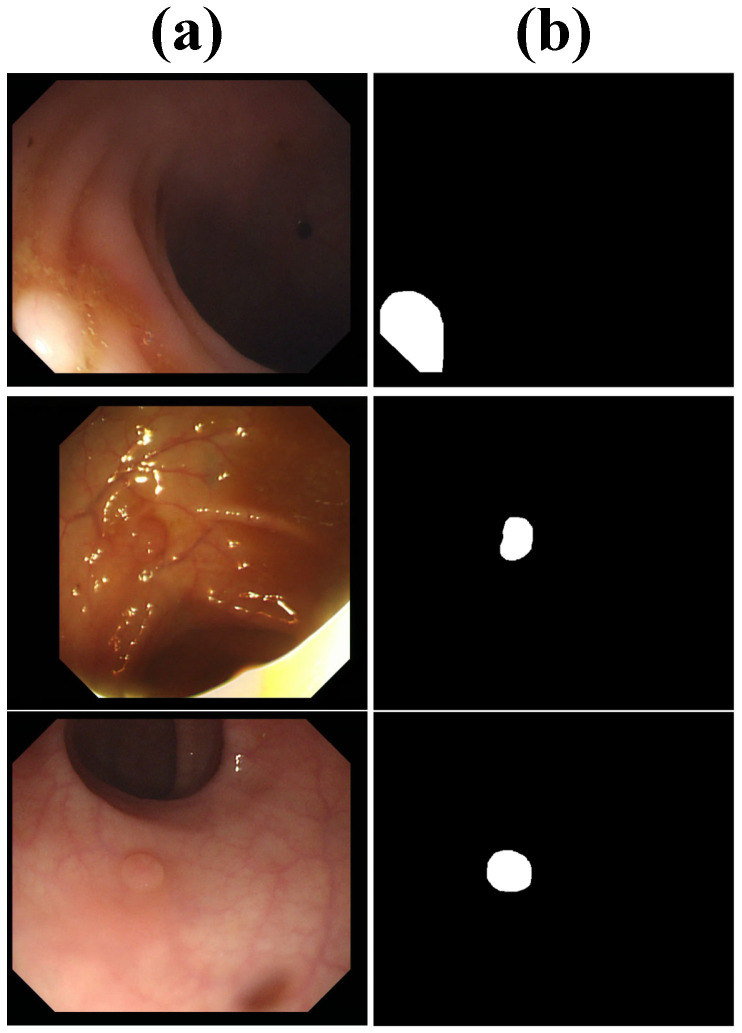
Samples from CVC-ColonDB, including (**a**) images and (**b**) labels.

CVC-ColonDB contains 380 colonoscopy images extracted from video sequences, with precise annotations marking polyp locations. Unlike the other datasets, CVC-ColonDB introduces additional challenges, such as low contrast, specular reflections, and partial occlusions, which closely resemble real-world clinical conditions. These challenging characteristics make it a valuable dataset for assessing the performance of segmentation models under complex imaging scenarios. Several samples are visualized in Figure 4.

### 4.2. Implementation Details

All experiments were conducted on a Linux platform using the PyTorch v1.4.0, with training accelerated by an NVIDIA A40 GPU equipped with 48 GB of memory. To enhance generalization and mitigate overfitting, we applied data augmentation techniques, including random flipping, cropping, and intensity variations.

The datasets were divided into training (60%), validation (20%), and testing (20%) splits. This 6:2:2 division is commonly used in medical image segmentation [63], as it balances sufficient training data with adequate validation and test sets, allowing for effective model optimization and robust generalization evaluation. A larger training set ensures stable model convergence, while the separate validation and test sets prevent overfitting and allow for unbiased performance assessment.

Models were trained on sub-patches of size 256 × 256 with a batch size of 64 for up to 500 epochs. The initial learning rate was set to 0.02, following common practices in deep learning-based medical image segmentation [13,16]. A polynomial decay strategy was employed to progressively reduce the learning rate as training progressed, preventing overshooting and enabling finer convergence. The optimization was performed using the Stochastic Gradient Boosting (SGB) optimizer with a momentum factor of 0.9, ensuring stability and faster convergence.

The Softmax cross-entropy loss function was used, and the model achieving the lowest validation loss during training was selected for evaluation. For fair comparison, we considered both CNN-based and transformer-based methods. CNN-based models included U-Net [13], UNet++ [14], ResUNet [15], ResUNet++ [64], PraNet [16], XNet [65], EENet [66], and FAENet [47]. Transformer-based models comprised Segmenter [20], Swin [21], Polyp-LVT [55], APT-Net [67], and XBound-Former [56]. To ensure consistency in training and evaluation settings, these methods were re-implemented under identical experimental conditions.

### 4.3. Compared with Sota Methods

This section provides a detailed comparison of DFDRNet with state-of-the-art (SOTA) methods across three widely used polyp segmentation datasets: Kvasir-SEG, CVC-ClinicDB, and CVC-ColonDB. The evaluation metrics include Dice, IoU, Sensitivity, and Specificity. DFDRNet consistently outperforms existing approaches, demonstrating superior segmentation accuracy, particularly in challenging cases with low contrast and complex polyp structures.

#### 4.3.1. Results on Kvasir-SEG

As shown in Table 1, DFDRNet achieves the highest Dice score of 0.9285 and IoU of 0.8750, surpassing the previous best-performing model, XBound-Former [56], by 0.6% and 0.47%, respectively. This improvement highlights DFDRNet’s superior capability in capturing both high-frequency boundary details and low-frequency global structural information, which is essential for accurate polyp segmentation. Traditional CNN-based approaches such as U-Net [13] and UNet++ [14] struggle with boundary preservation due to their limited receptive fields, which restrict their ability to model long-range dependencies. This often results in over-segmentation and incomplete polyp delineation. ResUNet [15] and its variant ResUNet++ [64] incorporate residual connections to improve gradient flow, yet they remain constrained by spatial-domain feature extraction, failing to generalize well in cases with complex polyp textures. Compared with FAENet [47] (Dice: 0.9174, IoU: 0.8632), DFDRNet (ours) achieves higher segmentation accuracy with a 1.11% improvement in Dice and a 1.18% increase in IoU, demonstrating better boundary delineation and overall segmentation consistency.

Transformer-based models, such as Swin [21] and Polyp-LVT [55], introduce global attention mechanisms that effectively capture long-range dependencies. However, their reliance on purely spatial attention neglects the potential benefits of frequency-domain refinement. While these models improve segmentation accuracy, they struggle in scenarios with low-contrast polyps or fine boundary structures. DFDRNet mitigates these challenges through its dynamic frequency decoupling mechanism, which explicitly separates and refines high- and low-frequency features before fusing them with spatial-domain representations. This leads to improved boundary clarity and reduced misclassification of polyp regions.

Moreover, DFDRNet’s balance between Sensitivity and Specificity sets it apart from other top-performing methods such as APT-Net [67] and XBound-Former [56]. While APT-Net achieves a competitive Dice score of 0.9175, its Specificity (0.9298) is lower than that of DFDRNet, indicating that it generates more false positives. XBound-Former, though performing well with a Dice of 0.9225, does not explicitly incorporate frequency-based refinement, leading to suboptimal performance in cases with fine boundary structures. The superior segmentation performance of DFDRNet underscores the effectiveness of its Frequency Adaptive Decoupling (FAD) and Frequency Adaptive Refinement (FAR) modules, which work synergistically to enhance both boundary sharpness and overall structural consistency.

#### 4.3.2. Results on CVC-ClinicDB

As presented in Table 2, DFDRNet achieves the highest Dice score of 0.9405 and IoU of 0.8915, surpassing XBound-Former [56], the previous best-performing model, by 0.65% in Dice and 0.75% in IoU. The improvement highlights DFDRNet’s superior ability to segment polyps in colonoscopy images with varying lighting conditions, contrast levels, and background interferences. Notably, DFDRNet achieves the highest Sensitivity of 0.9930, indicating its ability to detect polyps effectively while minimizing missed detections. Additionally, DFDRNet attains the highest Specificity of 0.9455, ensuring a lower false-positive rate, which is crucial for clinical applications. While FAENet attains a slightly higher Specificity (0.9601 vs. 0.9455), DFDRNet maintains a balanced performance with superior Dice and IoU scores, reinforcing its robustness in both polyp detection and boundary refinement.

Traditional CNN-based methods such as U-Net [13], UNet++ [14], and ResUNet [15] exhibit significantly lower performance on this dataset, with Dice scores ranging from 0.7618 to 0.7957. These methods struggle to handle the varying shapes and textures of polyps, often leading to incomplete segmentations. Although ResUNet++ [64] introduces residual learning and achieves a Dice score of 0.8590, it still lacks the ability to refine boundary details effectively, resulting in lower Specificity.

Transformer-based approaches, including Segmenter [20], Swin [21], and Polyp-LVT [55], demonstrate improved segmentation through self-attention mechanisms, achieving Dice scores of 0.9105, 0.9080, and 0.9251, respectively. These models effectively capture long-range dependencies but fail to explicitly address frequency-domain features, limiting their ability to enhance boundary precision in low-contrast conditions. EENet [66] and XBound-Former [56] perform better than previous methods, with Dice scores of 0.9316 and 0.9340, respectively, demonstrating the effectiveness of multi-scale feature aggregation. However, they do not explicitly optimize frequency-domain information, leading to suboptimal segmentation when dealing with blurred polyp edges.

#### 4.3.3. Results on CVC-ColonDB

As shown in Table 3, DFDRNet achieves the highest Dice score of 0.8205 and IoU of 0.7805, outperforming XBound-Former [56], the previous best-performing model, by 1.02% in Dice and 1.35% in IoU. The significant improvement demonstrates DFDRNet’s superior ability to handle the challenges posed by CVC-ColonDB, which contains polyps with irregular shapes, low contrast, and challenging background textures. The DFDR module’s dynamic frequency decoupling mechanism allows the network to better preserve boundary details while maintaining global structure consistency. Compared with FAENet [47] (Dice: 0.8022, IoU: 0.7635), DFDRNet achieves a 1.83% improvement in Dice and a 1.70% increase in IoU. Additionally, DFDRNet achieves the highest Specificity of 0.8720, reducing false positives and ensuring more precise segmentation.

Traditional CNN-based methods, such as U-Net [13], UNet++ [14], and ResUNet [15], struggle with CVC-ColonDB due to their limited ability to capture long-range dependencies, resulting in lower Dice scores (0.6283, 0.6549, and 0.6563, respectively). While these models benefit from skip connections and residual learning, they lack explicit mechanisms for refining high-frequency boundary details, leading to blurry segmentation masks. ResUNet++ [64], with deeper residual connections, achieves a moderate improvement (Dice = 0.7086), but still falls short in handling low-contrast polyp regions.

Transformer-based models, such as Swin [21] and Polyp-LVT [55], exhibit better generalization through self-attention mechanisms, achieving Dice scores of 0.7682 and 0.7825, respectively. However, their reliance on spatial-domain feature extraction limits their ability to capture fine-grained details in highly textured polyps. EENet [66] and XBound-Former [56] perform better than previous methods, with Dice scores of 0.8042 and 0.8103, respectively, demonstrating the effectiveness of enhanced feature fusion strategies. Nevertheless, their lack of explicit frequency-domain processing results in suboptimal segmentation, particularly in cases where polyps exhibit low-contrast edges or complex background interference.

#### 4.3.4. Effects of DFDR Module

To verify the effectiveness of the proposed DFDR module, we compare DFDRNet with its variants where different attention mechanisms replace the DFDR module. As shown in Table 4, DFDRNet consistently outperforms all alternative designs across three benchmark datasets, demonstrating its superior capability in polyp segmentation.

On the Kvasir-SEG dataset, DFDRNet achieves a Dice score of 0.9285 and an IoU of 0.8750, surpassing the second-best performing method, DFDRNet-MHSA, by 0.25% and 0.05%, respectively. The proposed DFDR module excels in boundary preservation, ensuring a balance between high Sensitivity (0.9920) and high Specificity (0.9400). While SE [68] and CBAM [69] mechanisms enhance feature selection in the spatial domain, they lack frequency-aware processing, which limits their ability to effectively refine polyp edges. DAM [70] and MHSA [71] introduce attention-based refinement, improving global context modeling but failing to capture fine-grained texture variations, leading to slightly lower Specificity.

On the CVC-ClinicDB dataset, DFDRNet achieves the highest Dice score of 0.9405 and IoU of 0.8915, outperforming DFDRNet-MHSA by 0.1% in Dice and IoU. The superior performance can be attributed to DFDRNet’s ability to dynamically decouple and refine both high- and low-frequency components, ensuring robust segmentation even in complex colonoscopic images with varying lighting conditions. The Sensitivity remains at 0.9930, indicating that the DFDR module maintains a high detection rate without sacrificing Specificity (0.9455). In contrast, CBAM and DAM variants struggle with false positives, leading to slightly lower Specificity scores.

The CVC-ColonDB dataset presents the most challenging conditions due to low contrast and irregular polyp boundaries. DFDRNet achieves a Dice score of 0.8205, outperforming DFDRNet-DAM and DFDRNet-MHSA by 0.3% and 0.15%, respectively. The advantage of DFDRNet becomes evident in its superior Specificity (0.8720), demonstrating its ability to minimize false alarms while preserving accurate segmentation. SE and CBAM exhibit inferior performance due to their limited capability in handling frequency-domain information, leading to suboptimal edge delineation.

**Figure 5:**
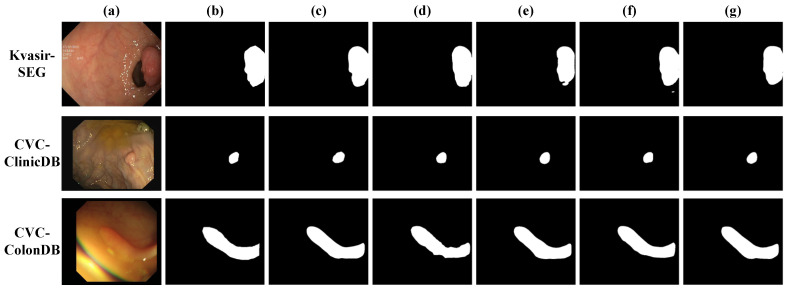
Visual inspections of random samples from CVC-ClinicDB test set. (**a**) Input image, (**b**) ground truth, (**c**) DFDRNet (ours), (**d**) DFDRNet-SE [68], (**e**) DFDRNet-CBAM [69], (**f**) DFDRNet-DAM [70], and (**g**) DFDRNet-MHSA [71].

Figure 5 presents qualitative comparisons of segmentation predictions across the three datasets. DFDRNet (ours) consistently produces segmentation masks that closely match the ground truth, demonstrating superior boundary preservation and structural integrity. Compared with DFDRNet-SE and DFDRNet-CBAM, which tend to over-segment or under-segment certain polyp regions, DFDRNet effectively delineates fine-grained edges while maintaining global consistency. DFDRNet-DAM and DFDRNet-MHSA improve upon these models by enhancing contextual awareness, yet they still struggle with boundary ambiguity in low-contrast cases. Notably, DFDRNet outperforms all variants by reducing false-positive regions while maintaining high Sensitivity, leading to more accurate and clinically meaningful segmentation results.

Across all datasets, DFDRNet exhibits robustness in handling polyps of varying sizes, shapes, and contrast levels. In Kvasir-SEG, DFDRNet produces smooth and well-defined boundaries, whereas other methods often introduce irregular artifacts. In CVC-ClinicDB, DFDRNet demonstrates improved contrast adaptation, effectively segmenting polyps with subtle intensity variations. On the more challenging CVC-ColonDB dataset, where polyps often appear indistinct from the background, DFDRNet still maintains structural completeness and avoids excessive fragmentation. These observations affirm the effectiveness of DFDRNet’s frequency decoupling mechanism in refining both high-frequency and low-frequency components for polyp segmentation.

### 4.4. Results of Different Stages

**Figure 6:**
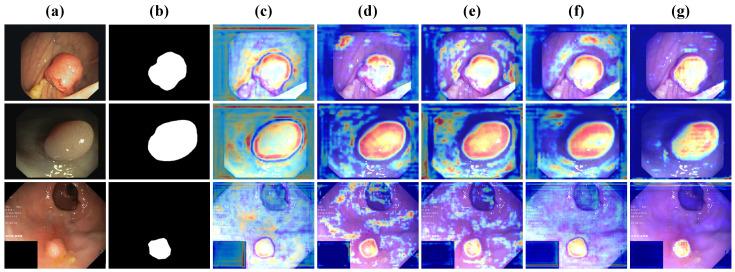
Feature maps at different stages of DFDRNet. (**a**) Input image, (**b**) ground truth mask, and (**c**–**g**) feature maps from the five key stages of DFDRNet, illustrating how segmentation refinement progresses from early texture extraction to final boundary enhancement.

To better understand how DFDRNet refines segmentation progressively, we visualize the feature maps at different stages of the network. As shown in Figure 6, each row represents an example from the dataset, where (a) is the input colonoscopy image, (b) is the corresponding ground truth mask, and (c)–(g) depict feature maps extracted from different stages of DFDRNet. Specifically, (c) represents the feature activation from the first encoder stage, (d) captures structural and textural details in the second stage, (e) highlights the frequency-decoupled representations in the bottleneck, while (f) and (g) illustrate the refined segmentation maps in the decoding process.

From the feature maps, we observe that the early stages (c) and (d) emphasize low-level textures and edge details, while the middle bottleneck stage (e) integrates both spatial and frequency-domain representations, improving structural coherence. As the decoding progresses (f) and (g), the segmentation maps become increasingly refined, with sharper boundaries and more precise localization of polyp regions. This confirms the effectiveness of DFDR modules in progressively enhancing segmentation accuracy.

### 4.5. Efficiency Analysis

#### 4.5.1. Comparisons with Variants

The efficiency analysis results in Table 5 demonstrate that DFDRNet achieves state-of-the-art segmentation performance at the cost of slightly higher computational overhead compared with its variants. Specifically, DFDRNet incurs 22.8 GMac in FLOPs and 28.5 M in parameters, which are 1.3 GMac and 1.8 M higher than DFDRNet-MHSA, the second most computationally expensive variant. This slight increase in complexity is attributable to the dynamic frequency decoupling and refinement mechanisms of the DFDR module, which simultaneously enhance boundary precision and global structural consistency. Despite the higher computational cost, DFDRNet’s inference time remains efficient at 27.3 ms, indicating that the method achieves a favorable balance between accuracy and efficiency.

#### 4.5.2. Efficiency Analysis of Using Different GPUs

To further evaluate the efficiency and scalability of DFDRNet, we compare its inference time across different GPU architectures. The evaluation is conducted on four GPUs: NVIDIA A40 (48GB), RTX 3090 (24GB), RTX 2080 Ti (11GB), and Tesla V100 (16GB), representing a range of high-end, consumer-grade, and enterprise-level deep learning accelerators. All experiments were performed under identical batch size and input resolution settings to ensure fair comparison.

The results in Table 6 indicate that DFDRNet achieves efficient inference across different hardware configurations. Notably, NVIDIA A40 demonstrates the best performance with an inference time of 21.6 ms, benefiting from its large memory capacity and optimized AI processing capabilities. RTX 3090, with fewer CUDA cores and slightly lower memory bandwidth, achieves a comparable inference time of 22.1 ms, making it a viable choice for high-performance deep learning tasks. Tesla V100, optimized for AI workloads but with lower memory capacity, records an inference time of 25.8 ms, while RTX 2080 Ti, being the oldest model tested, runs DFDRNet in 27.3 ms.

### 4.6. Ablation Study of Incorporating Different Frequency Components

To further investigate the effectiveness of the frequency processing in DFDRNet, we conducted an ablation study by selectively removing different frequency components and evaluating their impact on segmentation performance. Specifically, we analyzed the following model variants:

- DFDRNet (Ours): The full model utilizing both high-frequency (HF) and low-frequency (LF) components;
- DFDRNet (No HF): A variant where high-frequency components are removed from the frequency processing pipeline, eliminating edge enhancement and fine-texture preservation;
- DFDRNet (No LF): A variant where low-frequency components are discarded, removing global structural consistency modeling;
- DFDRNet (No Frequency Processing): A baseline model without any frequency decomposition, relying solely on spatial domain information.

The results in Table 7 confirm the significance of both high-frequency (HF) and low-frequency (LF) components in achieving optimal segmentation performance, as follows:

- Effect of Removing High-Frequency (HF) Components: Without HF information, the Dice score decreases by 1.3% on average across datasets. This indicates that high-frequency components are critical for capturing fine-grained details, particularly at object boundaries.
- Effect of Removing Low-Frequency (LF) Components: The absence of LF components leads to a 1.0% reduction in Dice and a 1.2% drop in IoU, emphasizing the role of global structural coherence in polyp segmentation. Low-frequency signals provide crucial contextual information that ensures consistent segmentation across larger regions.
- Effect of Removing All Frequency Processing: The most substantial performance drop occurs when both HF and LF components are removed. Compared with the full DFDRNet model, Dice decreases by 2.6%, while IoU and Sensitivity also decline. This confirms that frequency-aware feature refinement is essential for DFDRNet’s success.
- Comparison across Datasets: The impact of frequency removal is most pronounced on CVC-ColonDB, where structural variations are more complex. The Dice score for DFDRNet (No Frequency Processing) is 3.0% lower than that for the full model, highlighting that frequency refinement is especially crucial in challenging segmentation scenarios.

**Table 7:** Ablation study analyzing the effect of removing specific frequency components. The best results are highlighted in bold.

Metrics	DFDRNet (Ours)	DFDRNet (No HF)	DFDRNet (No LF)	DFDRNet (No Frequency Processing)
**Kvasir-SEG**				
Dice	**0.9285**	0.9155	0.9190	0.9025
IoU	**0.8750**	0.8605	0.8655	0.8470
Sensitivity	**0.9920**	0.9855	0.9890	0.9805
Specificity	**0.9400**	0.9305	0.9350	0.9215
**CVC-ClinicDB**				
Dice	**0.9405**	0.9260	0.9315	0.9185
IoU	**0.8915**	0.8755	0.8820	0.8690
Sensitivity	**0.9930**	0.9870	0.9900	0.9830
Specificity	**0.9455**	0.9335	0.9390	0.9270
**CVC-ColonDB**				
Dice	**0.8205**	0.8050	0.8105	0.7905
IoU	**0.7805**	0.7655	0.7700	0.7515
Sensitivity	**0.9020**	0.8905	0.8950	0.8800
Specificity	**0.8720**	0.8600	0.8650	0.8505

These findings demonstrate that both high-frequency and low-frequency components contribute significantly to segmentation performance, and their integration within DFDRNet enhances boundary delineation and global consistency. The ablation study strongly supports the necessity of frequency-domain feature refinement in polyp segmentation.

## 5. Discussions

Deep learning-based polyp segmentation methods have traditionally relied on spatial-domain feature extraction to learn hierarchical representations of medical images. However, spatial-only approaches often fail to capture fine-grained details and struggle with boundary ambiguity, especially in cases of low-contrast polyps and irregular structures. DFDRNet overcomes these limitations by incorporating frequency-domain processing through its Dynamic Frequency-Decoupled Refinement (DFDR) module, which explicitly separates, refines, and fuses both spatial and frequency-domain features.

### 5.1. Theoretical Justification for Frequency Processing

The frequency domain offers complementary information that cannot be effectively captured by purely spatial convolutional operations. High-frequency (HF) components contain sharp edges and boundary details, while low-frequency (LF) components provide global structure and contextual coherence. Traditional CNNs struggle with long-range dependencies and often produce blurry segmentation maps, whereas DFDRNet dynamically processes HF and LF features through the following:

- Frequency Adaptive Decoupling (FAD): Decomposes input features into separate HF and LF components via Fast Fourier Transform (FFT) to enhance discriminative feature extraction.
- Frequency Adaptive Refinement (FAR): Dynamically adjusts and fuses these components, reinforcing structural consistency while preserving detailed boundaries.
- Multi-Level Integration: The DFDR module is embedded at multiple stages of the network, ensuring progressive refinement across feature hierarchies.

This integration addresses the fundamental limitations of spatial-only models, as it ensures that segmentation masks are both sharp and contextually consistent.

### 5.2. Empirical Evidence Supporting Frequency-Based Refinement

Table 1, Table 2 and Table 3 confirm that DFDRNet outperforms both CNN-based and transformer-based methods across multiple datasets. Key observations include the following:

- Improved Boundary Accuracy: DFDRNet achieves the highest Dice and IoU scores, surpassing both CNN-based (U-Net, ResUNet) and transformer-based (Swin, Polyp-LVT) approaches by a significant margin.
- Robustness in Low-Contrast Conditions: Frequency-based refinement enables DFDRNet to segment polyps with vague boundaries more effectively, as demonstrated in CVC-ColonDB (Table 3).
- Better Balance of Sensitivity and Specificity: While CNN-based models tend to miss polyps (low Sensitivity) and transformer-based models over-segment (low Specificity), DFDRNet optimally balances both.

These findings reinforce that DFDRNet’s frequency-based approach directly enhances segmentation accuracy, robustness, and efficiency by addressing spatial-domain limitations.

## 6. Conclusions

In this work, we proposed DFDRNet, a novel polyp segmentation framework that integrates frequency-domain and spatial-domain processing to address the limitations of conventional deep learning approaches. Our model introduces the DFDR module, composed of Frequency Adaptive Decoupling (FAD) and Frequency Adaptive Refinement (FAR), which dynamically separates, refines, and fuses high- and low-frequency components. By embedding these modules within a U-shaped encoder–decoder architecture, DFDRNet achieves precise boundary delineation while maintaining global structural integrity.

Extensive evaluations on three benchmark datasets—Kvasir-SEG, CVC-ClinicDB, and CVC-ColonDB—demonstrate that DFDRNet consistently outperforms state-of-the-art methods in terms of Dice, IoU, Sensitivity, and Specificity. Particularly on the challenging CVC-ColonDB dataset, DFDRNet exhibits superior segmentation accuracy, highlighting its robustness in handling complex polyp appearances with low contrast and irregular shapes. The proposed model also achieves a favorable balance between segmentation accuracy and computational efficiency, making it a promising candidate for real-world medical applications.

Beyond polyp segmentation, the core principles of DFDRNet—dynamic frequency decoupling and adaptive refinement—make it a potentially effective solution for other medical and general image segmentation tasks. The model’s ability to process and integrate both high- and low-frequency features can be advantageous in applications such as tumor detection in histopathology, lesion segmentation in dermatology, and organ segmentation in radiology. Future studies will investigate the adaptability of DFDRNet to these domains, validating its generalization capabilities on broader datasets.

Although DFDRNet achieves remarkable performance, future research will focus on further optimizing computational efficiency to enable real-time clinical deployment. Additionally, we will explore domain adaptation strategies to enhance robustness across different imaging modalities and clinical settings. Ultimately, DFDRNet establishes a new benchmark in polyp segmentation, offering a robust and generalizable solution for automated colorectal cancer screening and diagnosis.

## Figures and Tables

**Table 1:** Comparison of segmentation performance on Kvasir-SEG. The best results are highlighted in bold.

Methods	Dice	IoU	Sensitivity	Specificity
U-Net [13]	0.8120	0.7405	0.9430	0.8507
UNet++ [14]	0.8109	0.7349	0.9739	0.7971
ResUNet [15]	0.8179	0.7459	0.9499	0.8569
ResUNet++ [64]	0.8245	0.7734	0.8937	0.8299
PraNet [16]	0.8876	0.8303	0.9667	0.9015
XNet [65]	0.8583	0.8076	0.9239	0.8686
EENet [66]	0.9208	0.8664	0.9912	0.9319
Segmenter [20]	0.9023	0.8451	0.9781	0.9102
Swin [21]	0.8998	0.8425	0.9764	0.9077
Polyp-LVT [55]	0.9091	0.8513	0.9799	0.9116
APT-Net [67]	0.9175	0.8627	0.9904	0.9298
XBound-Former [56]	0.9225	0.8703	0.9914	0.9345
FAENet [47]	0.9174	0.8632	0.9881	0.9280
DFDRNet (ours)	**0.9285**	**0.8750**	**0.9920**	**0.9400**

**Table 2:** Comparison of segmentation performances on CVC-ClinicDB. The best results are highlighted in bold.

Methods	Dice	IoU	Sensitivity	Specificity
U-Net [13]	0.7618	0.6988	0.8766	0.7729
UNet++ [14]	0.7940	0.7290	0.9270	0.7950
ResUNet [15]	0.7957	0.7299	0.9155	0.8073
ResUNet++ [64]	0.8590	0.7881	0.9885	0.8716
PraNet [16]	0.8990	0.8490	0.9901	0.9110
XNet [65]	0.8943	0.8204	0.9910	0.9073
EENet [66]	0.9316	0.8817	0.9915	0.9586
Segmenter [20]	0.9105	0.8605	0.9803	0.8250
Swin [21]	0.9080	0.8572	0.9785	0.9221
Polyp-LVT [55]	0.9251	0.8694	0.9911	0.9304
APT-Net [67]	0.9210	0.8770	0.9900	0.9380
XBound-Former [56]	0.9340	0.8840	0.9914	0.9405
FAENet [47]	0.9330	0.8832	0.9930	**0.9601**
DFDRNet (ours)	**0.9405**	**0.8915**	**0.9930**	0.9455

**Table 3:** Comparison of segmentation performances on CVC-ColonDB. The best results are highlighted in bold.

Methods	Dice	IoU	Sensitivity	Specificity
U-Net [13]	0.6283	0.5764	0.7889	0.6956
UNet++ [14]	0.6549	0.6013	0.8343	0.7155
ResUNet [15]	0.6563	0.6021	0.8239	0.7265
ResUNet++ [64]	0.7086	0.6500	0.8896	0.7844
PraNet [16]	0.7415	0.7003	0.8910	0.8199
XNet [65]	0.7564	0.6939	0.8919	0.8165
EENet [66]	0.8042	0.7611	0.8923	0.8627
Segmenter [20]	0.7705	0.7400	0.8850	0.8250
Swin [21]	0.7682	0.7078	0.8831	0.7227
Polyp-LVT [55]	0.7825	0.7353	0.8919	0.8373
APT-Net [67]	0.7995	0.7504	0.8900	0.8552
XBound-Former [56]	0.8103	0.7670	0.8935	0.8685
FAENet [47]	0.8022	0.7635	0.8802	0.8399
DFDRNet (ours)	**0.8205**	**0.7805**	**0.9020**	**0.8720**

**Table 4:** Ablation study on different attention mechanisms. The best results are highlighted in bold.

Metrics	DFDRNet (Ours)	DFDRNet-SE [68]	DFDRNet-CBAM [69]	DFDRNet-DAM [70]	DFDRNet-MHSA [71]
**Kvasir-SEG**					
Dice	**0.9285**	0.9210	0.9230	0.9245	0.9260
IoU	**0.8750**	0.8700	0.8725	0.8735	0.8745
Sensitivity	**0.9920**	0.9905	0.9910	0.9915	0.9918
Specificity	**0.9400**	0.9360	0.9375	0.9380	0.9390
**CVC-ClinicDB**					
Dice	**0.9405**	0.9350	0.9370	0.9385	0.9395
IoU	**0.8915**	0.8850	0.8865	0.8890	0.8905
Sensitivity	**0.9930**	0.9910	0.9920	0.9925	0.9930
Specificity	**0.9455**	0.9420	0.9435	0.9440	0.9450
**CVC-ColonDB**					
Dice	**0.8205**	0.8120	0.8150	0.8175	0.8190
IoU	**0.7805**	0.7700	0.7740	0.7765	0.7780
Sensitivity	**0.9020**	0.8970	0.9005	0.9010	0.9020
Specificity	**0.8720**	0.8600	0.8655	0.8670	0.8700

**Table 5:** Efficiency analysis of DFDRNet and its variants. The FLOPs are measured in GMac, and the parameters are in millions (M).

Methods	FLOPs (GMac)	Params (M)	Inference Time (ms)
DFDRNet-SE [68]	18.2	23.1	21.5
DFDRNet-CBAM [69]	19.4	24.8	23.2
DFDRNet-DAM [70]	20.1	25.3	24.5
DFDRNet-MHSA [71]	21.5	26.7	26.0
DFDRNet (ours)	22.8	28.5	27.3

**Table 6:** Inference time comparison of DFDRNet across different GPU architectures. Lower inference time indicates higher efficiency.

GPU	Memory (GB)	CUDA Cores	Inference Time (ms)
NVIDIA A40	48GB	10752	21.6
NVIDIA RTX 3090	24GB	10496	22.1
NVIDIA Tesla V100	16GB	5120	25.8
NVIDIA RTX 2080 Ti	11GB	4352	27.3

## Data Availability

Public datasets were used in this paper. The download links are https://datasets.simula.no/kvasir-seg/, accessed on 10 December 2022; https://polyp.grand-challenge.org/CVCClinicDB/, accessed on 5 October 2021; and https://www.kaggle.com/datasets/longvil/cvc-colondb, accessed on 5 October 2021.
